# Acoustic Features Differentiate Subtypes of Cognitive Impairment in a Digital Cognitive Assessment

**DOI:** 10.1093/geroni/igaf122.3523

**Published:** 2025-12-31

**Authors:** Tanya Talkar, Daniel Schulman, Connor Higgins, Kan Kawabata, Sean Tobyne

**Affiliations:** Linus Health, Boston, Massachusetts, United States; Linus Health, Boston, Massachusetts, United States; Linus Health, Boston, Massachusetts, United States; Linus Health, Boston, Massachusetts, United States; Linus Health, Boston, Massachusetts, United States

## Abstract

The Digital Assessment of Cognition (DAC) is a brief, automated, remote-capable cognitive assessment that includes an animal naming task, a backward digit span test (BDST), and a 6-word Philadelphia Verbal Learning Test (PVLT). Patient speech produced during these tasks is automatically analyzed, yielding a rich set of acoustic and prosodic features that are potentially informative of cognitive impairment. We leverage a recently-developed clustering model that differentiates subtypes of mild cognitive impairment (MCI) and dementia using DAC outputs, and examine between-cluster differences in acoustic features in a dataset (N = 1188) gathered from the Apheleia-001 pre-screener study. Across all tasks, speaking rate, pause duration (mean and std.), pause percentage, and reaction time increase with worsening cognitive impairment, while we see a decrease in voice quality (cepstral peak prominence and harmonic to noise ratio). We particularly see an increase in reaction time during PVLT immediate recall trials with the presence of increased cognitive impairment, but an even more pronounced difference between dementia and MCI subtypes during the delayed recall trial. Similarly, speaking rate showed significant differences between subtypes during the delayed recall task. Our results suggest that response latency related features, and voice quality, can be affected within tasks due to the presence of cognitive impairment, with differences in subtypes likely due to differences in the domains of impairment. We conclude that speech-based features are informative of characterizing both cognitive impairment overall, and of specific subtypes of MCI and dementia. Future work can leverage these features to improve detection of cognitive impairment subtypes.

